# Phase 1b/2 study of the liposomal formulation of eribulin (E7389-LF) in combination with nivolumab: Results from the phase 2 esophageal cancer cohort

**DOI:** 10.1038/s44276-024-00066-6

**Published:** 2024-09-04

**Authors:** Takashi Oshima, Sachiko Yamamoto, Hisato Kawakami, Tomoki Makino, Akihito Kawazoe, Toshiki Masuishi, Takahiro Tsushima, Motohiro Hirao, Masahiro Tsuda, Kaori Hino, Noboru Yamamoto, Hiroki Hara, Shota Kaname, Daiko Matsuoka, Yohei Otake, Keisuke Yasuda, Takao Takase, Shuya Takashima, Taro Semba, Akira Ooki

**Affiliations:** 1 Kanagawa Cancer Center, Yokohama, Japan; 2 Osaka International Cancer Institute, Osaka, Japan; 3 Kindai University Faculty of Medicine, Osakasayama, Japan; 4 Osaka University Graduate School of Medicine, Suita, Japan; 5 National Cancer Hospital East, Kashiwa, Japan; 6 Aichi Cancer Center Hospital, Nagoya, Japan; 7 Shizuoka Cancer Centre, Shizuoka, Japan; 8 NHO, Osaka, Japan; 9 Hyogo Cancer Center, Hyogo, Japan; 10 NHO Shikoku Cancer Center, Ehime, Japan; 11 National Cancer Center Hospital, Tokyo, Japan; 12 Saitama Cancer Center, Saitama, Japan; 13 Ono Pharmaceutical Co., Ltd., Osaka, Japan; 14 Eisai Co., Ltd., Tokyo, Japan; 15 Eisai Co., Ltd., Ibaraki, Japan; 16 The Cancer Institute Hospital of Japanese Foundation for Cancer Research, Tokyo, Japan

## Abstract

**Background:**

Esophageal cancer is one of the most common types of cancer in Japan. Herein, we report the efficacy and safety of E7389-LF plus the immune checkpoint inhibitor, nivolumab, from the esophageal cancer cohort of the phase 2 part of Study 120.

**Methods:**

Eligible patients received E7389-LF 2.1 mg/m^2^ plus nivolumab 360 mg intravenously Q3W. The primary objective was to evaluate the objective response rate (ORR); other objectives included safety, progression-free survival (PFS), and overall survival (OS).

**Results:**

Of the 35 Japanese patients enrolled, 7 (20.0%) had a partial response as their best overall response, and 14 (40.0%) had stable disease. The ORR was 20.0% (95% CI 8.4–36.9). The duration of response was 5.6 months (95% CI 1.7–not estimable [NE]). The median PFS was 2.81 months (95% CI 1.31–4.17). The median OS was not reached (95% CI 6.54 months–NE). The most common treatment-emergent adverse events were neutropenia (65.7%), pyrexia (60.0%), and leukopenia (57.1%). Select plasma endothelial cell markers levels increased from day 1 of cycle 1 and changes were pronounced between days 8–15 of each cycle.

**Conclusions:**

E7389-LF plus nivolumab showed antitumor activity in patients with unresectable and pretreated esophageal cancer and should be evaluated further in a broader population.

**Clinical Trial Registration:**

NCT04078295.

## Introduction

Esophageal cancer (EGC) is one of the most common types of cancer in Japan. Per Globocan 2020, it was the 11th most common cancer by incidence and the 10th most common by mortality [1]; the majority of these cases were classified as esophageal squamous cell carcinoma [2]. Standard treatment for patients with unresectable locally advanced, recurrent, or metastatic disease usually involves combination therapy, typically comprised of a platinum drug with an immune checkpoint inhibitor (ICI) such as nivolumab [3], or double-ICI therapy [3, 4]. Second-line therapy is dependent on first-line therapy but usually features an ICI and/or a taxane [3].

In the ATTRACTION-3 trial, treatment with nivolumab was associated with significantly improved overall survival over chemotherapy in patients with advanced esophageal cancer refractory or intolerant to fluoropyrimidine-based and platinum-based chemotherapy [5]. Previously, the combination of eribulin mesylate with an anti-programmed cell death protein 1 (anti-PD-1) antibody showed antitumor activity in a preclinical mouse model [6].

E7389-LF is a liposomal formulation of eribulin mesylate [7]; it has previously shown efficacy and vascular remodeling effects in patients with advanced solid tumors [8]. In a preclinical study, E7389-LF had superior immunomodulatory activity compared with eribulin mesylate and had antitumor activity in combination with an anti-PD-1 antibody [9].

The open-label phase 1b/2 Study 120 was conducted to evaluate the potential antitumor activity of E7389-LF in combination with nivolumab in patients with various solid tumors. The phase 1b part evaluated the dosing and safety of E7389-LF plus nivolumab in 25 Japanese patients with solid tumors and determined a recommended phase 2 dose (RP2D) of 2.1 mg/m^2^ E7389-LF plus 360 mg nivolumab every 3 weeks [10]. Phase 2 assessed efficacy and safety in cohorts of patients with gastric cancer, esophageal cancer, and small-cell lung cancer. Herein, we report the efficacy and safety of E7389-LF plus nivolumab from the esophageal cancer cohort of the phase 2 part of Study 120.

## Methods

### Study design and patients

Japanese patients with unresectable and measurable esophageal cancer who were previously treated with 1 line of combination therapy (including a platinum agent and a fluoropyrimidine, without a taxane, as a first-line therapy) were enrolled to the esophageal cancer cohort of the phase 2 part of Study 120 (NCT04078295). Eligible patients could have either squamous cell carcinoma or adenocarcinoma and received E7389-LF in combination with nivolumab dosed at the RP2D of the phase 1b part, E7389-LF 2.1 mg/m^2^ plus nivolumab 360 mg administered intravenously every 3 weeks. In the case of treatment-emergent adverse events (TEAEs) related to treatment, E7389-LF could be dose-reduced in consecutive steps to 1.7 mg/m^2^, 1.1 mg/m^2^, and 0.8 mg/m^2^; dose reductions of nivolumab were not permitted.

Enrolled patients were required to have at least 1 measurable lesion based on Response Evaluation Criteria in Solid Tumors version 1.1 (RECIST v1.1); lesions previously treated with radiotherapy or locoregional therapies must have shown evidence of progressive disease to be deemed a measurable lesion. Additionally, patients needed to have an Eastern Cooperative Oncology Group performance status (ECOG PS) of 0 or 1. Patients were excluded if they previously had an immune-related adverse event (irAE) of grade ≥3 from, or requiring discontinuation of: (1) any anti-PD-1, anti-programmed cell death ligand 1 (anti-PD-L1), anti-PD-L2, anti-CD137, or anti-CTLA-4 antibody; (2) any other antibody or drug specifically targeting T-cell costimulation or checkpoint; (3) cancer vaccine therapy.

This study was conducted in accordance with the standard operating procedures of the sponsor, which were based on the Principles of the World Medical Association Declaration of Helsinki, all applicable Japanese Good Clinical Practices and regulations, and the Pharmaceutical Affairs Law for studies conducted in Japan.

### Objectives

The primary objective of the phase 2 part of Study 120 was to evaluate the objective response rate (ORR); key secondary objectives included assessment of safety and progression-free survival (PFS). Exploratory objectives included assessments of overall survival (OS) and disease control rate (DCR), as well as evaluation of potential biomarkers.

Tumor responses were assessed every 6 weeks by the investigators (both the principal investigator and sub-investigators) per RECIST v1.1. Safety assessments consisted of monitoring and recording all adverse events, and grading was conducted according to Common Terminology Criteria for Adverse Events–National Cancer Institute (CTCAE-NCI) version 5.0. Patients were allowed to receive prophylactic granulocyte colony-stimulating factor (peg-GCSF) for the prevention of febrile neutropenia.

Blood samples for use in biomarker analyses were collected predose on days 1, 8, and 15 of cycles 1 and 2, and on day 1 of subsequent cycles (until cycle 9), and off-treatment. Plasma biomarkers were investigated using AngiogenesisMAP®, Multiplex, and Simoa systems. PD-L1 expression in tumor samples was evaluated based on the PD-L1 combined positive score (CPS, %, based on PD-L1 expression in viable tumor cells and immune cells) and the tumor proportion score (TPS, %, based on PD-L1 expression in viable tumor cells) using an approved immunohistochemical 28-8 pharmDx assay (Agilent Dako, Agilent Technologies, Santa Clara, CA).

### Assessments and statistical analyses

ORR was calculated as the proportion of patients with a complete response or partial response. The sample size was calculated based on a Bayesian posterior probability of over 85% beyond the threshold (20%) for ORR; this required enrollment of a minimum of 32 patients. DCR was calculated as the proportion of patients with a complete response, partial response, or stable disease (for ≥5 weeks after first dosing). 95% Confidence intervals (CIs) for ORR and DCR were calculated using Clopper-Pearson’s exact method. Complete and partial responses required confirmation by a subsequent assessment of the response at least 28 days later. For PFS and OS, medians were estimated by the Kaplan–Meier method, and 95% CIs were calculated based on the Greenwood formula. Estimates for survival follow-up time were calculated in the same way as the Kaplan–Meier estimates of OS but with the meanings of ʻcensorʼ and ʻeventʼ status indicators reversed. Statistical analyses were performed using SAS software (version 9.4, SAS Institute, Cary, NC).

The effect of E7389-LF in combination with nivolumab on soluble biomarkers was assessed. Plasma biomarkers were analyzed if ≥75% of the samples demonstrated levels above the lower limit of quantification. Percent changes from cycle 1 day 1 were assessed using the 1-sample Wilcoxon signed-rank test. For the percent change in biomarker analyses using all markers, *P*-values were adjusted using the Benjamini–Hochberg procedure for false discovery rate control, with the number of biomarkers analyzed at each time point.

## Results

### Patients

Among the 35 Japanese patients who enrolled in the esophageal cancer cohort of the phase 2 expansion part, 31 were male (Table 1). Patient median age was 69.0 years. All patients had squamous cell carcinoma and had received 1 anticancer medication regimen (excluding regimens in the adjuvant/neoadjuvant setting). Detailed data on previous therapies received are listed in Supplementary Table 1. By the data cutoff date (May 31, 2022), 5 patients (14.3%) were still undergoing treatment. Discontinuations occurred in 30 patients (85.7%); the primary reasons for discontinuation were as follows: 25 (71.4%) patients discontinued due to disease progression, 4 (11.4%) due to an adverse event, and 1 (2.9%) due to patient preference. The median follow-up time was 10.2 months (95% CI 8.4–12.0). During survival follow up, patients received subsequent anticancer medications that are detailed in Supplementary Table 1 and Supplementary Fig. 1.

**Table 1:** Patient Demographics and Baseline Characteristics.

Characteristic	Patients (*N* = 35)
Median age, years (range)	69.0 (47–85)
**Sex,** ***n*** **(%)**	
Male	31 (88.6)
Female	4 (11.4)
**ECOG PS,** ***n*** **(%)**	
0	21 (60.0)
1	14 (40.0)
Median weight, kg (range)	57.0 (38.3–81.5)
Median BSA, m^2^ (range)	1.635 (1.31–1.92)
Liver metastasis, *n* (%)	9 (25.7)
**Baseline PD-L1 CPS,** ***n*** **(%)**	
≥1	22 (62.9)
<1	8 (22.9)
≥5	13 (37.1)
<5	17 (48.6)
Missing	5 (14.3)
**Number of previous anticancer medication regimens (excluding adjuvant/neo-adjuvant),** ***n*** **(%)**	
1	35 (100)^a^

^a^7 Patients (20.0%) also received 1 line of adjuvant/neoadjuvant therapy, and thus had 2 lines of prior anticancer therapy.

*BSA* body surface area, *CPS* combined positive score, *ECOG PS* Eastern Cooperative Oncology Group performance status, *PD-L1* programmed cell death ligand 1.

### Efficacy

Of the 35 patients enrolled, 7 (20.0%) had a partial response as their best overall response, and 14 (40.0%) had stable disease (Table 2). The ORR was 20.0% (95% CI 8.4–36.9); DCR was 60.0% (95% CI 42.1–76.1) (Table 2), and the median duration of response was 5.6 months (95% CI 1.7–not estimable [NE]) (Supplementary Table 2). Among the 8 patients with a PD-L1 CPS of <1 at baseline, 1 had a partial response (ORR 12.5%; 95% CI 0.3–52.7); 6 of the 22 patients with a CPS of ≥1 had a partial response (ORR 27.3%; 95% CI 10.7–50.2). Among the 10 patients with a PD-L1 TPS of <1 at baseline, 2 had a partial response (ORR 20.0%; 95% CI 2.5–55.6); 5 of the 20 patients with a TPS of ≥1 had a partial response (ORR 25.0%; 95% CI 8.7–49.1) (Table 2). Responses by PD-L1 CPS and PD-L1 TPS of <5 and ≥5 are included in Supplementary Table 2.

**Table 2:** Tumor Responses in All Patients and by PD-L1 CPS and PD-L1 TPS.

**Parameter**	**PD-L1 CPS** < **1 (*n*** = **8)**	**PD-L1 CPS** ≥ **1 (*n*** = **22)**	**PD-L1 TPS** < **1 (*n*** = **10)**	**PD-L1 TPS** ≥ **1 (*n*** = **20)**	**Overall (*N*** = **35)**
**Best overall response,** ***n*** **(%)**					
PR	1 (12.5)	6 (27.3)	2 (20.0)	5 (25.0)	7 (20.0)
SD	2 (25.0)	10 (45.5)	2 (20.0)	10 (50.0)	14 (40.0)
PD	4 (50.0)	6 (27.3)	5 (50.0)	5 (25.0)	13 (37.1)
Unknown/ Not evaluable	1 (12.5)	0	1 (10.0)	0	1 (2.9)
**ORR,** ***n*** **(%)**	1 (12.5)	6 (27.3)	2 (20.0)	5 (25.0)	7 (20.0)
95% CI	0.3–52.7	10.7–50.2	2.5–55.6	8.7–49.1	(8.4–36.9)
**DCR,** ***n*** **(%)**	3 (37.5)	16 (72.7)	4 (40.0)	15 (75.0)	21 (60.0)
95% CI	8.5–75.5	49.8–89.3	12.2–73.8	50.9–91.3	42.1–76.1
**Median PFS, months (95% CI)**^**a**^	1.35 (0.56–2.83)	3.96 (1.18–5.52)	1.35 (0.56–6.77)	3.96 (1.18–5.52)	2.81 (1.31–4.17)
6-month PFS rate, % (95% CI)	14.3 (0.7–46.5)	22.7 (8.3–41.4)	22.2 (3.4–51.3)	20.0 (6.2–39.3)	20.2 (8.7–35.0)
Patients with PFS events, n (%)^b^	6 (75.0)	21 (95.5)	8 (80.0)	19 (95.0)	31 (88.6)
**Median OS, months (95% CI)**	6.57 (4.57–NE)	NR (5.49–NE)	6.57 (1.71–NE)	NR (5.49–NE)	NR (6.54–NE)
6-month OS rate, % (95% CI)	75.0 (31.5–93.1)	72.7 (49.1–86.7)	70.0 (32.9–89.2)	75.0 (50.0–88.7)	71.4 (53.4–83.5)
Patients with OS events, n (%)^c^	5 (62.5)	8 (36.4)	6 (60.0)	7 (35.0)	15 (42.9)

^a^PFS was assessed per RECIST v1.1 by investigators; ^b^PFS events include progressive disease or death; ^c^OS events were deaths.

*CI* confidence interval, *CPS* combined positive score, *DCR* disease control rate, *NE* not estimable, *NR* not reached, *ORR* objective response rate, *OS* overall survival, *PD* progressive disease, *PD-L1* programmed cell death ligand 1, *PFS* progression-free survival, *PR* partial response, *RECIST v1.1* Response Evaluation Criteria in Solid Tumors version 1.1, *SD* stable disease, *TPS* tumor proportion score.

**Fig. 1:**
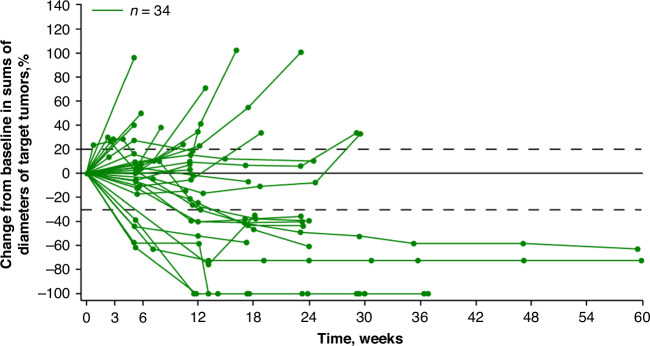
Changes in sums of diameters in target lesions from baseline over time. Patients with disease progression are included. Patients could continue to receive study drugs beyond disease progression if they had investigator-assessed clinical benefits and were tolerating the study drugs.

Generally, partial responses were sustained through dose reductions of E7389-LF (ie, 1.7 and 1.1 mg/m^2^) (Supplementary Fig. 2). Changes in the size of target lesions over time are shown in Fig. 1.

**Fig. 2:**
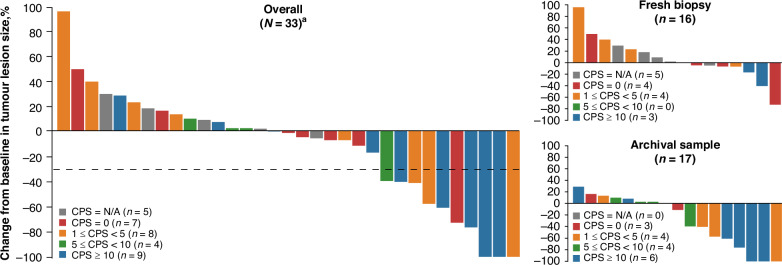
Change in tumor lesion size from baseline to postbaseline Nadir by PD-L1 CPS. Changes are reported in evaluable patients overall, evaluable patients who had a fresh biopsy sample available, and evaluable patients who were assessed based on an archival tissue sample. ^a^Of the 35 patients enrolled, 1 was excluded from the waterfall plot as they had a tumor response of progressive disease (the target lesion was not evaluable) at their first assessment, and another patient was excluded because of tumor size data post-drug administration. CPS combined positive score, N/A not available, PD-L1 programmed cell death ligand 1.

Patients with higher PD-L1 CPS at baseline tended to have a greater maximum degree of tumor shrinkage. However, tumor shrinkage was observed in patients with both high and low PD-L1 scores at baseline (Fig. 2).

**Fig. 3:**
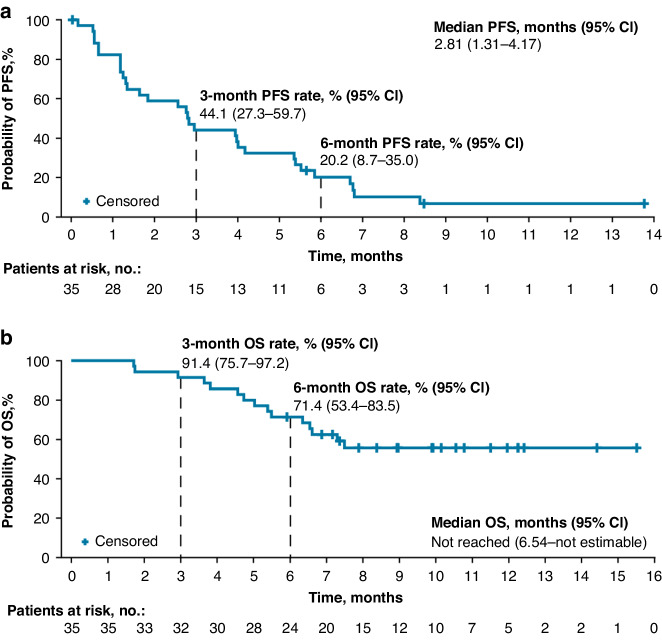
Kaplan–Meier survival curves. **a** PFS per RECIST v1.1 by Investigator Assessment. **b** OS. CI confidence interval, OS overall survival, PFS progression-free survival, RECIST v1.1 Response Evaluation Criteria In Solid Tumors version 1.1.

Overall, the median PFS was 2.81 months (95% CI 1.31–4.17), and the 6-month PFS rate was 20.2% (95% CI 8.7–35.0) (Fig. 3a). Among the 8 patients with a PD-L1 CPS of <1 at baseline, the median PFS was 1.35 months (95% CI 0.56–2.83) and the 6-month PFS rate was 14.3% (95% CI 0.7–46.5) (Table 2). Among the 22 patients with a PD-L1 CPS of ≥1, the median PFS was 3.96 months (95% CI 1.18–5.52) and the 6-month PFS rate was 22.7% (95% CI 8.3–41.4) (Table 2). Similarly, PFS outcomes appeared greater in the subgroup of patients who had a PD-L1 CPS of ≥5 versus <5 (Supplementary Table 2).

The median OS was not reached (95% CI 6.54 months–NE), and the 6-month OS rate was 71.4% (95% CI 53.4–83.5) (Fig. 3b). Among the 8 patients with a PD-L1 CPS of <1 at baseline, the median OS was 6.57 months (95% CI 4.57–NE) and the 6-month OS rate was 75.0% (95% CI 31.5–93.1) (Table 2). In the 22 patients with a PD-L1 CPS of ≥1, the median OS was not reached (95% CI 5.49 months–NE) and the 6-month OS rate was 72.7% (95% CI 49.1–86.7) (Table 2). OS outcomes among patients who had a PD-L1 CPS of <5 and ≥5 are listed in Supplementary Table 2.

### Safety

Treatment-related TEAEs of any grade and of grade ≥3 severity occurred in 94.3% and 80.0% of patients, respectively (Table 3). The most common treatment-related TEAEs of any grade were neutropenia (65.7%), leukopenia (57.1%), and decreased appetite (45.7%); the most common treatment-related TEAEs of grade ≥3 severity were neutropenia (54.3%), leukopenia (34.3%), and febrile neutropenia (22.9%). A summary of the most frequent treatment-related TEAEs by grade is shown in Supplementary Table 3.

**Table 3:** Summary of Treatment-Related TEAEs.

	Patients (*N* = 35)	
	Any Grade	Grade ≥ 3
Any treatment-related TEAE, *n* (%)	33 (94.3)	28 (80.0)
**Treatment-related TEAEs occurring in** > **10% of patients,** ***n*** **(%)**		
Neutropenia	23 (65.7)	19 (54.3)
Leukopenia	20 (57.1)	12 (34.3)
Decreased appetite	16 (45.7)	2 (5.7)
Pyrexia	13 (37.1)	0
Alopecia	12 (34.3)	0
Stomatitis	12 (34.3)	2 (5.7)
Thrombocytopenia	11 (31.4)	2 (5.7)
Fatigue	9 (25.7)	0
Febrile neutropenia	8 (22.9)	8 (22.9)
Nausea	8 (22.9)	0
Lymphopenia	7 (20.0)	5 (14.3)
Rash	7 (20.0)	0
Anemia	6 (17.1)	1 (2.9)
Malaise	6 (17.1)	1 (2.9)
Pruritus	6 (17.1)	0
Infusion-related reaction	5 (14.3)	0
Hypothyroidism	4 (11.4)	0
Dysgeusia	4 (11.4)	0

*TEAE* treatment-emergent adverse event.

All patients had a TEAE of any cause, and 33 (94.3%) had a TEAE of grade ≥3 severity (Supplementary Table 4). The most common TEAEs overall were neutropenia (65.7%), pyrexia (60.0%), and leukopenia (57.1%). No grade 5 TEAEs were reported. TEAEs led to dose reduction of E7389-LF in 17 patients (48.6%) and withdrawal of either E7389-LF or nivolumab in 5 patients (14.3%). TEAEs that led to withdrawal were pneumonia (*n* = 2), traumatic hemothorax (*n* = 1), acute kidney injury (*n* = 1), and pneumonitis (*n* = 1).

In patients who received peg-GCSF, the absolute neutrophil counts (ANCs) increased on cycle 1 day 8 and remained elevated in cycle 2 day 1 compared with cycle 1 day 1 (Supplementary Fig. 3a). In patients who did not receive peg-GCSF, ANCs decreased on cycle 1 day 8 but had recovered before the next dose of study treatment was administered (cycle 2 day 1) (Supplementary Fig. 3b). Reductions in ANC did not affect the next administration in the Q3W regimen. Additionally, none of the 9 patients who received prophylactic peg-GCSF during cycle 1 had febrile neutropenia; all 8 cases of febrile neutropenia occurred in 26 patients who did not receive prophylactic peg-GCSF.

### Biomarker and immune phenotype analyses

Of the 78 plasma biomarkers tested, 47 passed the criteria for analysis. Plasma endothelial cell markers collagen IV, TIE2 (tyrosine kinase immunoglobulin and epidermal growth factor homology domains 2, also known as TEK [TEK receptor tyrosine kinase]), ICAM1 (intercellular adhesion molecule 1), and PECAM1 (platelet endothelial cell adhesion molecule 1) increased from cycle 1 day 1 (Supplementary Fig. 4**;** Supplementary Table 5). Changes in endothelial markers were pronounced between days 8–15 of each cycle; generally, these changes were sustained through day 1 of each consecutive cycle. Plasma interferon gamma (IFNγ) and IFNγ-related markers, MIG (monokine induced by gamma interferon; also known as CXCL9 [c-x-c motif chemokine ligand 9]) and IP-10 (interferon gamma-induced protein 10, also known as CXCL10), also increased (Supplementary Fig. 4).

During cycle 1, changes in the levels of most biomarkers were the greatest on day 8. Changes in several biomarkers, including collagen IV and MIG were even more apparent after the second dose of study treatment. Further study is required to determine the significance of endothelial and IFNγ-related biomarkers in esophageal cancer.

## Discussion

Generally, E7389‐LF in combination with nivolumab had antitumor activity in patients with esophageal cancer. Reductions in the size of tumor lesions were observed regardless of PD-L1 status; however, patients with a CPS of ≥1 appeared to benefit particularly. Notably, patients with a PD-L1 CPS of ≥5 had an ORR of 23.1%.

Acknowledging the limitations of cross-study comparisons, the efficacy of E7389-LF in combination with nivolumab was consistent with the efficacy of nivolumab monotherapy in the ATTRACTION-3 study [5, 11]. The ORR (20.0%) was similar to nivolumab monotherapy seen in ATTRACTION-3 (Primary analysis: 19% [5]; Japanese subgroup: 22.4% [11]), and the DCR in this study (60.0%) was notable compared with that of nivolumab in the overall population (37%) and of the Japanese subgroup (41.1%). It should be noted that comparisons between studies are complicated by differences in sample size, as well as the fact that our study included a single arm and was not stratified.

Median OS was not reached in our trial. Although this limits comparison to other trials, it is notable that OS was not reached (in the overall population, as well as the PD-L1 CPS ≥ 1 and TPS ≥ 1 subgroups) after a median follow-up of 10.2 months; in ATTRACTION-3, the median OS of the nivolumab arm (and of the PD-L1 CPS ≥ 1 subgroup) was 10.9 months in the primary analysis [5]. Further, in the Japanese subgroup of ATTRACTION-3, the median OS of nivolumab was 13.4 months, and 12.75 months in those with a PD-L1 CPS of ≥1 [11]. Thus, OS of E7389-LF in combination with nivolumab should be analyzed at a later date.

No new safety signals were observed compared to the known profiles of each monotherapy [12, 13], and the incidence of treatment-related leukopenia and neutropenia were similar to the phase 1b part of this study [10]. Myelosuppression was reversible, as shown by the reduction and rebound of ANCs within the first cycle. Additionally, febrile neutropenia could be avoided through prophylactic use of peg-GCSF at cycle 1.

Apparent changes in plasma endothelial cell/vasculature and IFNγ-related biomarkers suggest that this treatment combination induces pharmacodynamic changes. These results are consistent with the phase 1b part of this study [10].

This study was limited by the small sample size of a narrow scope of patients; only a single arm of Japanese patients with esophageal squamous cell carcinoma partook in this cohort, of whom few had biopsy samples available for the analysis of immune phenotypes. Additionally, given the emergence of ICI therapies as first-line therapy for esophageal cancer (per the recent approval of nivolumab plus platinum doublet-therapy or ipilimumab for unresectable, advanced esophageal squamous cell carcinoma [4, 14]), there is an emerging need for later-line therapies that retain antitumor activity in the presence of ICI resistance. Unfortunately, no patients who received prior ICI therapy were enrolled in this study. However, in a preclinical investigation, E7389-LF showed immunomodulatory activity and the combination therapy, E7389-LF plus anti–PD-1 antibody, showed promising antitumor activity by improving the tumor microenvironment via vascular remodeling, which enhanced the activity of the cancer immunotherapy in the 4T1 breast cancer mouse model [9]. In addition to this preclinical study, the combination E7389-LF plus nivolumab showed notable efficacy in patients who were pretreated with ICI in another cohort (small cell lung cancer) of Study 120 [15].

Taken together, these results show that the combination of E7389-LF plus nivolumab may be a suitable option for patients with unresectable and pretreated esophageal cancer and should be evaluated further in a broader patient body, including those who received an ICI as prior therapy.

## Supplementary information

Supplementary Material

## Data Availability

The data will not be available for sharing at this time as the data are commercially confidential. However, Eisai will consider written requests to share the data on a case-by-case basis.
