# Supplementary material for: Phase 1b/2 study of the liposomal formulation of eribulin (E7389-LF) in combination with nivolumab: Results from the phase 2 esophageal cancer cohort
Source: BJC Rep. 2024 Sep 4;2:66. doi: 10.1038/s44276-024-00066-6 (PMC11523933; doi:10.1038/s44276-024-00066-6)
Supplement: Supplementary file 1 — Supplementary Material [file 44276_2024_66_MOESM1_ESM.docx]

# **Supplementary Material**

## **Supplementary Table 1.** Previous and Subsequent Anticancer Therapies

| **Category** | **Patients (N=35)** |
| --- | --- |
| **Number of patients with any previous systemic chemotherapy, n (%)** | 35 (100) |
| Fluorouracil | 34 (97.1) |
| Cisplatin | 28 (80.0) |
| Levofolinic acid | 6 (17.1) |
| Oxaliplatin | 6 (17.1) |
| Gimeracil; Oteracil; Tegafur | 2 (5.7) |
| Investigational drug | 2 (5.7) |
| Carboplatin | 1 (2.9) |
| Fluorouracil; Folinic acid; Oxaliplatin | 1 (2.9) |
| **Number of anticancer medication during survival follow-up,**  **n (%)** |  |
| 1 | 13 (37.1) |
| 2 | 5 (14.3) |
| 3 | 0 |
| 4 | 0 |
| **Anti-cancer medication during survival follow-up, n (%)** | **18 (51.4)** |
| Paclitaxel | 12 (34.3) |
| Fluorouracil | 5 (14.3) |
| Levofolinic acid | 3 (8.6) |
| Oxaliplatin | 3 (8.6) |
| Cisplatin | 2 (5.7) |
| Docetaxel | 2 (5.7) |
| Pembrolizumab | 2 (5.7) |
| Futibatinib | 2 (5.7) |
| Nivolumab | 2 (5.7) |
| Amivantamab | 1 (2.9) |
| Enfortumab vedotin | 1 (2.9) |

## **Supplementary Table 2.** Efficacy Assessments of Patients With a PD-L1 CPS of <5 or ≥5 and a PD-L1 TPS of <5 or ≥5

|  | **PD-L1 CPS** | | **PD-L1 TPS** | | **Overall**  **(N=35)** |
| --- | --- | --- | --- | --- | --- |
| **Parameter** | **Patients with**  **PD-L1 CPS <5  (n = 17)** | **Patients with**  **PD-L1 CPS ≥5  (n = 13)** | **Patients with**  **PD-L1 TPS <5  (n = 22)** | **Patients with**  **PD-L1 TPS ≥5  (n = 8)** |  |
| **Best overall response, n (%)** |  |  |  |  |  |
| PR | 4 (23.5) | 3 (23.1) | 5 (22.7) | 2 (25.0) | 7 (20.0) |
| SD | 3 (17.6) | 9 (69.2) | 7 (31.8) | 5 (62.5) | 14 (40.0) |
| PD | 9 (52.9) | 1 (7.7) | 9 (40.9) | 1 (12.5) | 13 (37.1) |
| Unknown/not evaluable | 1 (5.9) | 0 | 1 (4.5) | 0 | 1 (2.9) |
| **ORR, n (%)**  95% CI | 4 (23.5)  6.8–49.9 | 3 (23.1) 5.0–53.8 | 5 (22.7)  7.8 ̶ 45.4 | 2 (25.0)  3.2 ̶ 65.1 | 7 (20.0)  8.4–36.9 |
| **DCR, n (%)**  95% CI | 7 (41.2)  18.4–67.1 | 12 (92.3) 64.0–99.8 | 12 (54.5)  32.2 ̶ 75.6 | 7 (87.5)  47.3 ̶ 99.7 | 21 (60.0)  42.1–76.1 |
| **Median duration of response^b^, months**  95% CI | 5.0  2.8–NE | 5.6  1.7–NE | 5.6  2.8–NE | NE  1.7–NE | 5.6  1.7–NE |
| **Median PFS, months (95% CI)^a^**  6-month PFS rate, % (95% CI)  Patients with PFS events, n (%)^b^ | 1.33 (0.66–3.98)  12.5 (2.1–32.8)  15 (88.2) | 4.17 (2.76–6.7)  30.8 (9.5–55.4)  12 (92.3) | 2.79 (1.18–3.98)  19.0 (5.9–37.7)  20 (90.9) | 4.85 (0.66–6.8)  25.0 (3.7–55.8)  7 (87.5) | 2.81 (1.31–4.17)  20.2 (8.7–35.0)  31 (88.6) |
| **Median OS, months (95% CI)**  6-month OS rate, % (95% CI)  Patients with OS events, n (%)^c^ | 6.6 (4.57–NE)  64.7 (37.7–82.3)  10 (58.8) | NR (7.29–NE)  84.6 (51.2–95.9)  3 (23.1) | 7.49 (5.03–NE)  68.2 (44.6–83.4)  11 (50.0) | NR (5.49–NE)  87.5 (38.7–98.1)  2 (25.0) | NR (6.54–NE)  71.4 (53.4–83.5)  15 (42.9) |

^a^Of the 5 patients with a missing PD-L1 CPS, 2 had SD and 3 had PD as their best overall response, ^b^The median duration of response was calculated using the Kaplan-Meier method for the duration from first documentation of CR or PR to PFS (event/censor) date among responders.

CI, confidence interval; CPS, combined positive score; CR, complete response; DCR, disease control rate; NE, not estimable; NR, not reached; ORR, objective response rate; PFS, progression-free survival; PD, progressive disease; PD-L1, programmed cell death ligand 1; PR, partial response; SD, stable disease; TPS, tumor proportion score.

## **Supplementary Table 3**. Summary of Treatment-Related Treatment-Emergent Adverse Events by CTCAE Grade

|  | **Patients (N = 35)** | | | | |
| --- | --- | --- | --- | --- | --- |
|  | **Grade 1** | **Grade 2** | **Grade 3** | **Grade 4** | **Grade 5** |
| **Any treatment-related TEAE, n (%)** | 0 | 5 (14.3) | 17 (48.6) | 11 (31.4) | 0 |
| **Treatment-related TEAEs occurring in >10% of patients, n (%)** | | | | | |
| Neutropenia | 0 | 4 (11.4) | 10 (28.6) | 9 (25.7) | 0 |
| Leukopenia | 0 | 8 (22.9) | 7 (20.0) | 5 (14.3) | 0 |
| Decreased appetite | 6 (17.1) | 8 (22.9) | 2 (5.7) | 0 | 0 |
| Pyrexia | 8 (22.9) | 5 (14.3) | 0 | 0 | 0 |
| Alopecia | 9 (25.7) | 3 (8.6) | 0 | 0 | 0 |
| Stomatitis | 3 (8.6) | 7 (20.0) | 2 (5.7) | 0 | 0 |
| Thrombocytopenia | 4 (11.4) | 5 (14.3) | 2 (5.7) | 0 | 0 |
| Fatigue | 3 (8.6) | 6 (17.1) | 0 | 0 | 0 |
| Febrile neutropenia | 0 | 0 | 8 (22.9) | 0 | 0 |
| Nausea | 5 (14.3) | 3 (8.6) | 0 | 0 | 0 |
| Lymphopenia | 0 | 2 (5.7) | 2 (5.7) | 3 (8.6) | 0 |
| Rash | 4 (11.4) | 3 (8.6) | 0 | 0 | 0 |
| Anemia | 0 | 5 (14.3) | 1 (2.9) | 0 | 0 |
| Malaise | 2 (5.7) | 3 (8.6) | 1 (2.9) | 0 | 0 |
| Pruritus | 4 (11.4) | 2 (5.7) | 0 | 0 | 0 |
| Infusion-related reaction | 3 (8.6) | 2 (5.7) | 0 | 0 | 0 |
| Hypothyroidism | 1 (2.9) | 3 (8.6) | 0 | 0 | 0 |
| Dysgeusia | 1 (2.9) | 3 (8.6) | 0 | 0 | 0 |

Adverse events were graded using CTCAE version 5.0.

CTCAE, common terminology criteria for adverse events; TEAE, treatment-emergent adverse event.

## **Supplementary Table 4**. Treatment-Emergent Adverse Events of Any Cause

|  | **Patients (N = 35)** | |
| --- | --- | --- |
|  | **Any Grade** | **Grade ≥3** |
| **Any TEAE, n (%)** | 35 (100) | 33 (94.3) |
| **TEAEs occurring in >10% of patients, n (%)** | | |
| Neutropenia | 23 (65.7) | 19 (54.3) |
| Pyrexia | 21 (60.0) | 1 (2.9) |
| Leukopenia | 20 (57.1) | 12 (34.3) |
| Decreased appetite | 19 (54.3) | 3 (8.6) |
| Stomatitis | 13 (37.1) | 2 (5.7) |
| Alopecia | 12 (34.3) | 0 |
| Thrombocytopenia | 11 (31.4) | 2 (5.7) |
| Fatigue | 10 (28.6) | 0 |
| Anemia | 9 (25.7) | 3 (8.6) |
| Nausea | 9 (25.7) | 0 |
| Febrile neutropenia | 8 (22.9) | 8 (22.9) |
| Pruritus | 8 (22.9) | 0 |
| Lymphopenia | 8 (22.9) | 6 (17.1) |
| Rash | 7 (20.0) | 0 |
| Malaise | 6 (17.1) | 1 (2.9) |
| Constipation | 6 (17.1) | 0 |
| Pneumonia | 6 (17.1) | 3 (8.6) |
| Aspartate aminotransferase increased | 5 (14.3) | 2 (5.7) |
| Diarrhea | 5 (14.3) | 0 |
| Dysgeusia | 4 (11.4) | 0 |
| Hypothyroidism | 4 (11.4) | 0 |
| Peripheral sensory neuropathy | 4 (11.4) | 0 |
| Weight decreased | 3 (8.6) | 0 |
| **TEAEs leading to:** | | |
| Dose reduction^a^ | 17 (48.6) | |
| Dose interruption^b^ | 16 (45.7) | |
| Withdrawal^b^ | 5 (14.3) | |

^a^Modification of E7389-LF; ^b^either study drug.

TEAE, treatment-emergent adverse event.

## **Supplementary Table 5.** Median Change in Biomarkers From Baseline

| **Biomarker** | **C1D8 (n = 35)** | | **C2D1 (n = 27)** | | **C2D8 (n = 26)** | | **C3D1 (n = 23)** | | **C4D1 (n = 20)** | | **C5D1 (n = 14)** | |
| --- | --- | --- | --- | --- | --- | --- | --- | --- | --- | --- | --- | --- |
|  | **Med % Δ from BL** | ***P*-value** | **Med % Δ from BL** | ***P*-value** | **Med % Δ from BL** | ***P*-value** | **Med % Δ from BL** | ***P*-value** | **Med % Δ from BL** | ***P*-value** | **Med % Δ from BL** | ***P*-value** |
| Angiopoietin 2 | 20.34 | **0.003** | 2.94 | 0.138 | 28.57 | **<0.001** | 0.00 | 0.571 | -4.41 | 0.701 | -7.56 | 0.497 |
| BAFF (B cell-activating factor) | 111.96 | **<0.001** | 68.85 | **<0.001** | 214.95 | **<0.001** | 96.08 | **<0.001** | 58.25 | **<0.001** | 48.38 | **0.002** |
| BLC (B lymphocyte chemoattractant) | 65.12 | **<0.001** | 25.71 | **<0.001** | 83.63 | **<0.001** | 43.75 | **<0.001** | 29.55 | **0.019** | 11.20 | 0.463 |
| Cancer antigen 15-3 | 7.50 | 0.037 | 10.00 | 0.226 | 18.21 | **0.017** | 17.14 | 0.078 | 16.90 | 0.241 | 12.54 | 0.502 |
| CA9 (carbonic anhydrase-9) | 115.79 | **<0.001** | -18.18 | 0.237 | 152.88 | **<0.001** | 15.00 | 0.131 | -11.97 | 0.984 | 0.00 | 0.622 |
| 6Ckine | -0.45 | 0.874 | 0.62 | 0.691 | -0.55 | 0.824 | 4.22 | 0.067 | -0.07 | 0.622 | 0.83 | 0.855 |
| Collagen IV | 97.62 | **<0.001** | 35.22 | **<0.001** | 216.89 | **<0.001** | 64.06 | **<0.001** | 40.35 | **<0.001** | 49.30 | **0.002** |
| Decorin | 27.27 | **<0.001** | -5.56 | 0.197 | 34.06 | **<0.001** | 0.00 | 1.000 | -10.51 | **0.019** | -14.93 | 0.066 |
| EGFR (epidermal growth factor receptor) | 3.33 | **0.005** | -7.89 | **0.007** | -4.76 | 0.979 | -10.64 | 0.057 | -12.13 | **0.008** | -9.29 | 0.151 |
| Endoglin | 2.00 | 0.536 | -2.33 | 0.372 | 10.01 | 0.053 | 7.69 | 0.184 | 1.55 | 0.335 | 6.62 | 1.000 |
| Fatty acid-binding protein, adipocyte | 106.25 | **<0.001** | 25.00 | **<0.001** | 126.14 | **<0.001** | 23.08 | **0.003** | 12.88 | 0.065 | -1.30 | 0.893 |
| Factor VII | -1.36 | 0.835 | 0.69 | 0.424 | -1.23 | 0.886 | -8.28 | 0.478 | 2.17 | 1.000 | -0.41 | 0.761 |
| Heparin-binding EGF-like growth factor | 25.00 | **<0.001** | 0.00 | **0.016** | 3.85 | 0.784 | -11.11 | **0.013** | -19.17 | **0.001** | -23.62 | **0.017** |
| Hepsin | 8.45 | **0.010** | 5.29 | 0.039 | 9.83 | **0.001** | 7.55 | **0.008** | 0.00 | 0.865 | -0.43 | 0.839 |
| HER2 (human epidermal growth factor receptor 2) | 2.44 | 0.591 | 0.00 | 0.610 | 13.41 | 0.026 | 6.00 | 0.284 | -6.00 | 0.632 | -12.92 | 0.677 |
| HGFR (hepatocyte growth factor receptor) | -1.19 | 0.913 | 3.66 | 0.032 | 7.19 | 0.057 | 6.67 | 0.105 | 2.36 | 0.202 | -5.93 | 0.296 |
| ICAM1 (intercellular adhesion molecule 1) | 27.78 | **<0.001** | 16.67 | **<0.001** | 33.33 | **<0.001** | 27.12 | **<0.001** | 21.69 | **0.011** | 27.13 | **0.017** |
| IFNγ (interferon gamma) | 176.60 | **<0.001** | 40.00 | **<0.001** | 228.18 | **<0.001** | 49.06 | **0.007** | 3.65 | 0.231 | 37.65 | 0.115 |
| IGFBP1 (insulin-like growth factor-binding protein 1) | 133.33 | **<0.001** | 81.25 | **<0.001** | 203.83 | **<0.001** | 80.00 | **<0.001** | 69.79 | **0.002** | 140.83 | **0.009** |
| IGFBP2 (insulin-like growth factor-binding protein 2) | -2.74 | 0.873 | 12.50 | 0.114 | 15.88 | 0.047 | 13.72 | 0.089 | 17.63 | 0.066 | 27.50 | 0.042 |
| IL13 (interleukin 13) | 0.00 | 0.552 | 60.00 | **<0.001** | 35.87 | **0.003** | 32.00 | **0.023** | -13.69 | 0.522 | -6.48 | 0.946 |
| IL18 (interleukin 18) | 72.19 | **<0.001** | 34.38 | **<0.001** | 114.34 | **<0.001** | 28.57 | **<0.001** | 18.92 | **0.004** | 14.81 | 0.194 |
| IL18BP (interleukin 18 binding protein) | 41.41 | **<0.001** | 25.00 | **<0.001** | 65.69 | **<0.001** | 26.67 | **<0.001** | 20.05 | **0.001** | 16.93 | **0.003** |
| IL8 (interleukin 8) | 125.00 | **<0.001** | 0.00 | 0.574 | 83.18 | **<0.001** | 0.00 | 0.681 | -15.60 | 0.841 | -17.67 | 0.670 |
| IP10 (interferon gamma-induced protein 10, CXCL10) | 157.59 | **<0.001** | 63.64 | **<0.001** | 223.68 | **<0.001** | 47.87 | **<0.001** | 61.60 | **0.001** | 52.72 | 0.042 |
| Kallikrein 5 | 0.00 | 0.255 | 6.17 | 0.560 | 28.66 | **<0.001** | 7.41 | 0.181 | 17.10 | 0.054 | -8.60 | 1.000 |
| Kallikrein 7 | -1.03 | 0.523 | -15.74 | 0.053 | -0.75 | 0.868 | -5.71 | 0.163 | -9.80 | 0.154 | 0.52 | 0.735 |
| MCP1 (monocyte chemotactic protein 1) | 118.80 | **<0.001** | -7.61 | 0.257 | 98.11 | **<0.001** | -24.79 | 0.058 | -23.67 | 0.245 | -27.52 | 0.078 |
| MIF (macrophage migration inhibitory factor) | 31.25 | **0.004** | 0.00 | 0.560 | 13.81 | 0.228 | 0.00 | 0.196 | -10.71 | 0.984 | -7.50 | 1.000 |
| MIG (monokine induced by gamma interferon, CXCL9) | 48.37 | **<0.001** | 74.26 | **<0.001** | 122.33 | **<0.001** | 132.08 | **<0.001** | 86.43 | **<0.001** | 75.04 | **0.005** |
| MIP1β (macrophage inflammatory protein-1 beta) | 81.28 | **<0.001** | 9.86 | 0.036 | 101.44 | **<0.001** | 7.25 | 0.176 | 7.55 | 0.277 | 2.08 | 0.455 |
| MIP3β (macrophage inflammatory protein-3 beta) | 55.10 | **<0.001** | 50.41 | **<0.001** | 82.06 | **<0.001** | 61.01 | **<0.001** | 24.14 | 0.027 | 37.18 | 0.042 |
| MMP3 (matrix metallopeptidase 3) | 34.02 | **<0.001** | 8.33 | **0.020** | 24.87 | **<0.001** | 9.89 | 0.272 | -2.38 | 0.535 | 8.97 | 0.326 |
| MMP9 (matrix metallopeptidase 9) | -38.97 | **<0.001** | 19.75 | **0.004** | -17.65 | 0.098 | 14.29 | 0.061 | 6.09 | 0.571 | -10.84 | 0.715 |
| PECAM1 (platelet endothelial cell adhesion molecule 1) | 15.00 | **<0.001** | 11.11 | **0.002** | 24.76 | **<0.001** | 22.92 | **<0.001** | 9.81 | **0.002** | 8.12 | **<0.001** |
| Prostasin | 4.19 | 0.102 | 2.33 | 0.225 | 8.04 | 0.060 | 7.66 | 0.067 | 0.60 | 0.729 | 5.10 | 0.670 |
| SCF (stem cell factor) | 0.00 | 0.039 | 0.00 | 0.390 | 0.00 | 0.153 | 0.00 | 0.300 | -10.91 | 0.263 | -26.20 | 0.455 |
| SDF1 (stromal cell-derived factor 1, CXCL12) | 22.26 | **<0.001** | 2.22 | 0.209 | 26.88 | **<0.001** | 2.60 | 0.108 | -3.56 | 0.756 | 0.79 | 0.542 |
| SPD (pulmonary surfactant-associated protein) | -26.67 | **<0.001** | 20.69 | **0.003** | -8.32 | 0.574 | 21.74 | 0.009 | 14.79 | 0.090 | 17.90 | 0.025 |
| TG (thyroglobulin) | 29.23 | **<0.001** | 17.86 | 0.044 | 42.53 | **<0.001** | 34.88 | **0.007** | 35.55 | **0.024** | 31.06 | 0.091 |
| TIE2 (tyrosine kinase immunoglobulin and epidermal growth factor homology domains 2, TEK) | 36.36 | **<0.001** | 18.75 | **<0.001** | 63.96 | **<0.001** | 18.75 | **<0.001** | 9.58 | 0.046 | 9.22 | 0.588 |
| TNC (tenascin-C) | 260.36 | **<0.001** | 66.97 | **<0.001** | 293.77 | **<0.001** | 55.86 | **<0.001** | 29.85 | **<0.001** | 34.52 | **0.002** |
| UPAR (urokinase-type plasminogen activator receptor) | 25.00 | **<0.001** | 18.18 | **<0.001** | 45.71 | **<0.001** | 30.00 | **<0.001** | 15.97 | 0.049 | -0.86 | 0.583 |
| VEGF (vascular endothelial growth factor) | 8.28 | **0.016** | -4.55 | 0.796 | -1.43 | 0.883 | -15.00 | 0.062 | -7.27 | 0.304 | -14.21 | 0.867 |
| VEGFD (vascular endothelial growth factor D) | 10.81 | **<0.001** | 5.97 | 0.480 | 0.80 | 0.351 | -1.43 | 0.718 | -2.19 | 0.571 | 15.73 | 0.035 |
| VEGFR2 (vascular endothelial growth factor receptor 2) | -8.89 | **0.014** | -4.26 | 0.578 | -6.26 | 0.036 | -7.89 | 0.589 | -7.17 | 0.156 | -6.25 | 0.196 |
| VEGFR3 (vascular endothelial growth factor receptor 3) | 5.41 | 0.121 | 9.43 | **<0.001** | 11.11 | **0.011** | 5.56 | 0.671 | -7.93 | 0.289 | -8.31 | 0.552 |

*P*-values shown are of Wilcoxon signed-rank tests; **bolded** values were significant after false-discovery-rate adjustment.

Δ, change; BL, baseline; C#D#, cycle # day #; CCL, C-C motif chemokine ligand; CXCL, C-X-C motif chemokine ligand; Med, median; TEK, TEK receptor tyrosine kinase.

## **Supplementary Figure 1**. Anti-Cancer Medication During Survival Follow-up


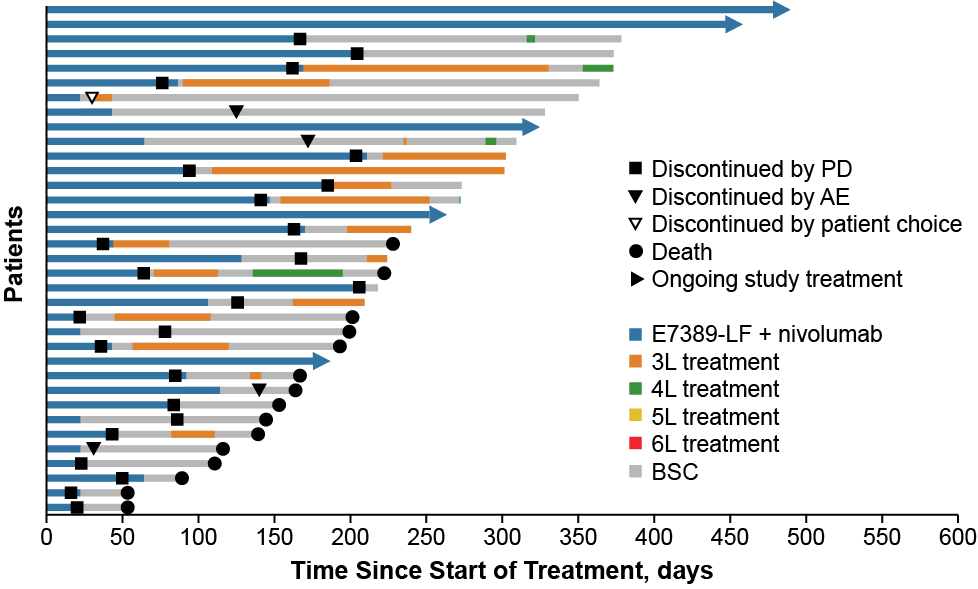


#L, number of lines of treatment; AE, adverse event; BSC, best supportive care; LF, liposomal formulation; PD, progressive disease

## **Supplementary Figure 2**. Patient Dosing and Responses Over Time


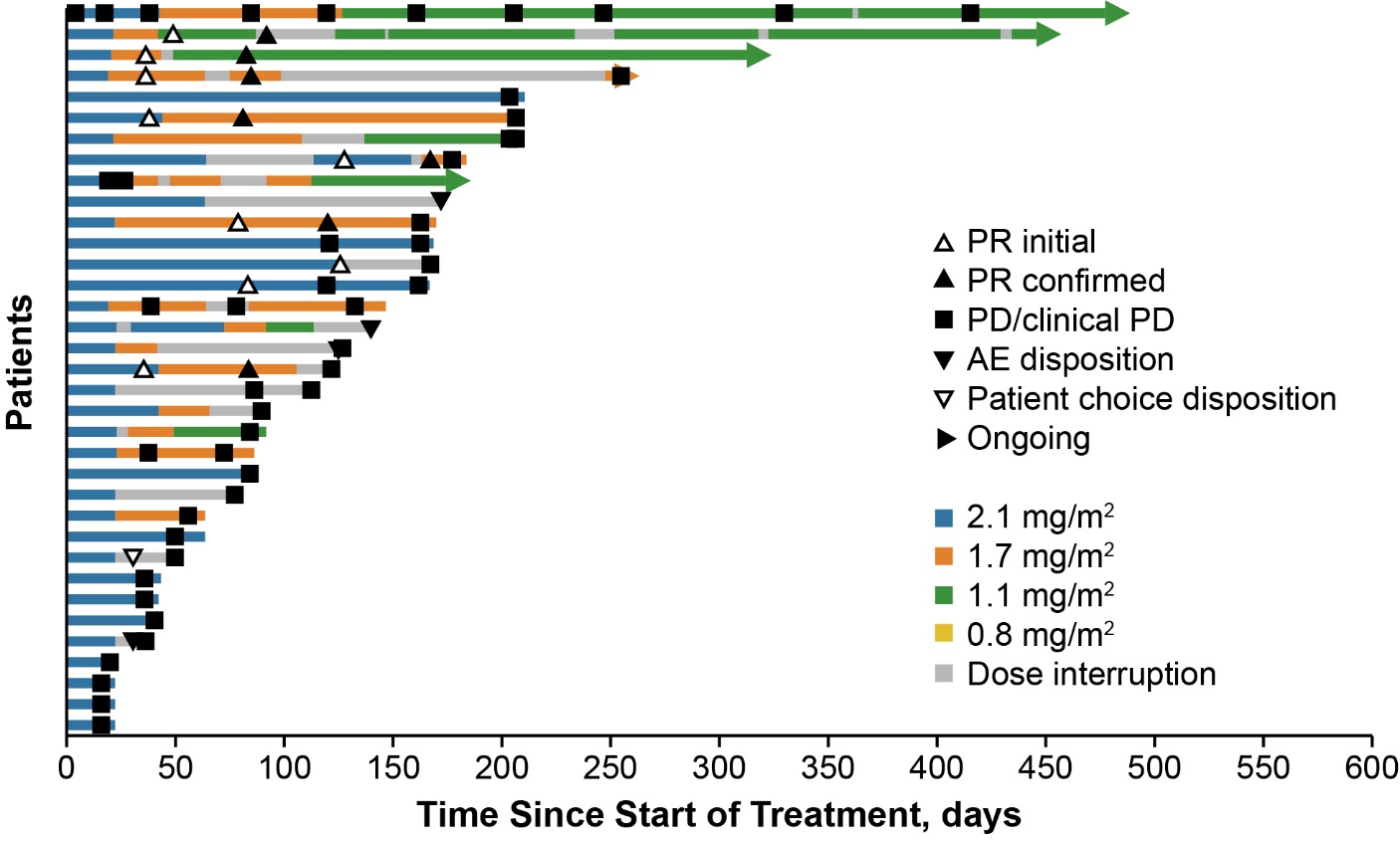


Patients could continue to receive study drugs beyond disease progression if they had investigator-assessed clinical benefit and were tolerating study drugs. This figure includes data from beyond disease progression.

AE, adverse event; PD, progressive disease; PR, partial response.

## **Supplementary Figure 3**. Absolute Neutrophil Counts by Visit


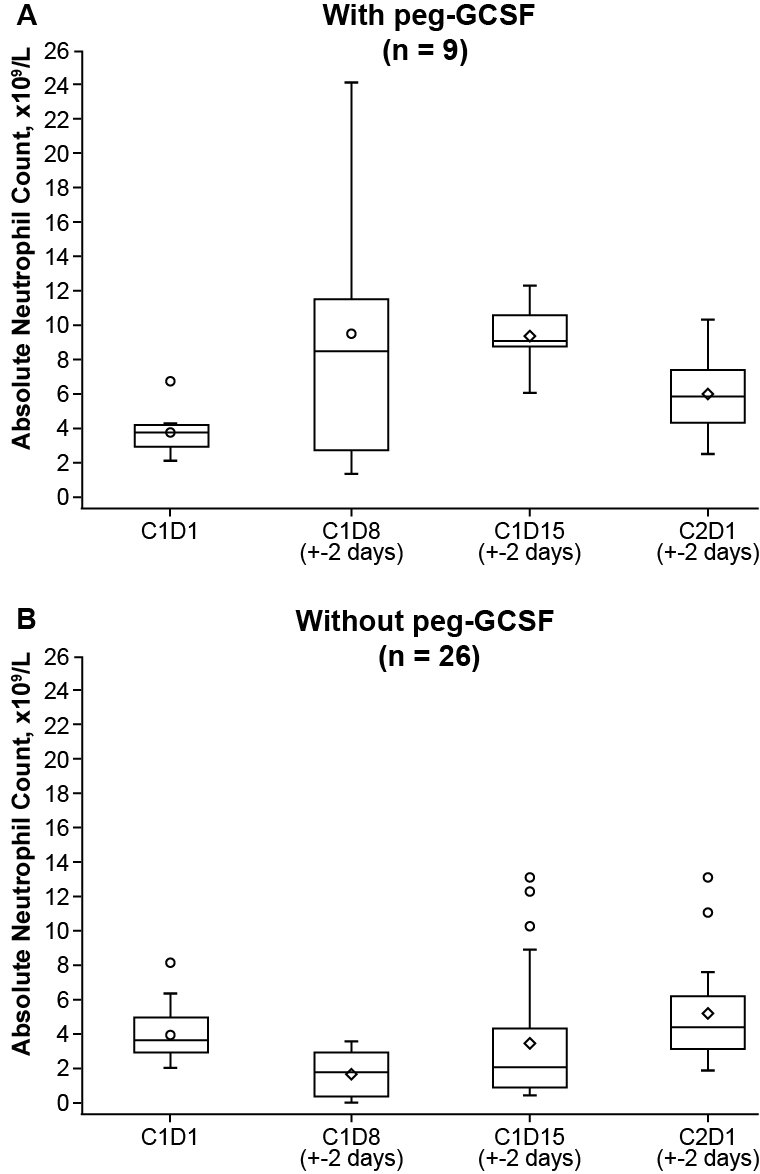


C#D#, cycle # day #; peg-GCSF, pegylated granulocyte colony-stimulating factor; Q3W, every 3 weeks.

## **Supplementary Figure 4.** Changes in Biomarker Levels From C1D1


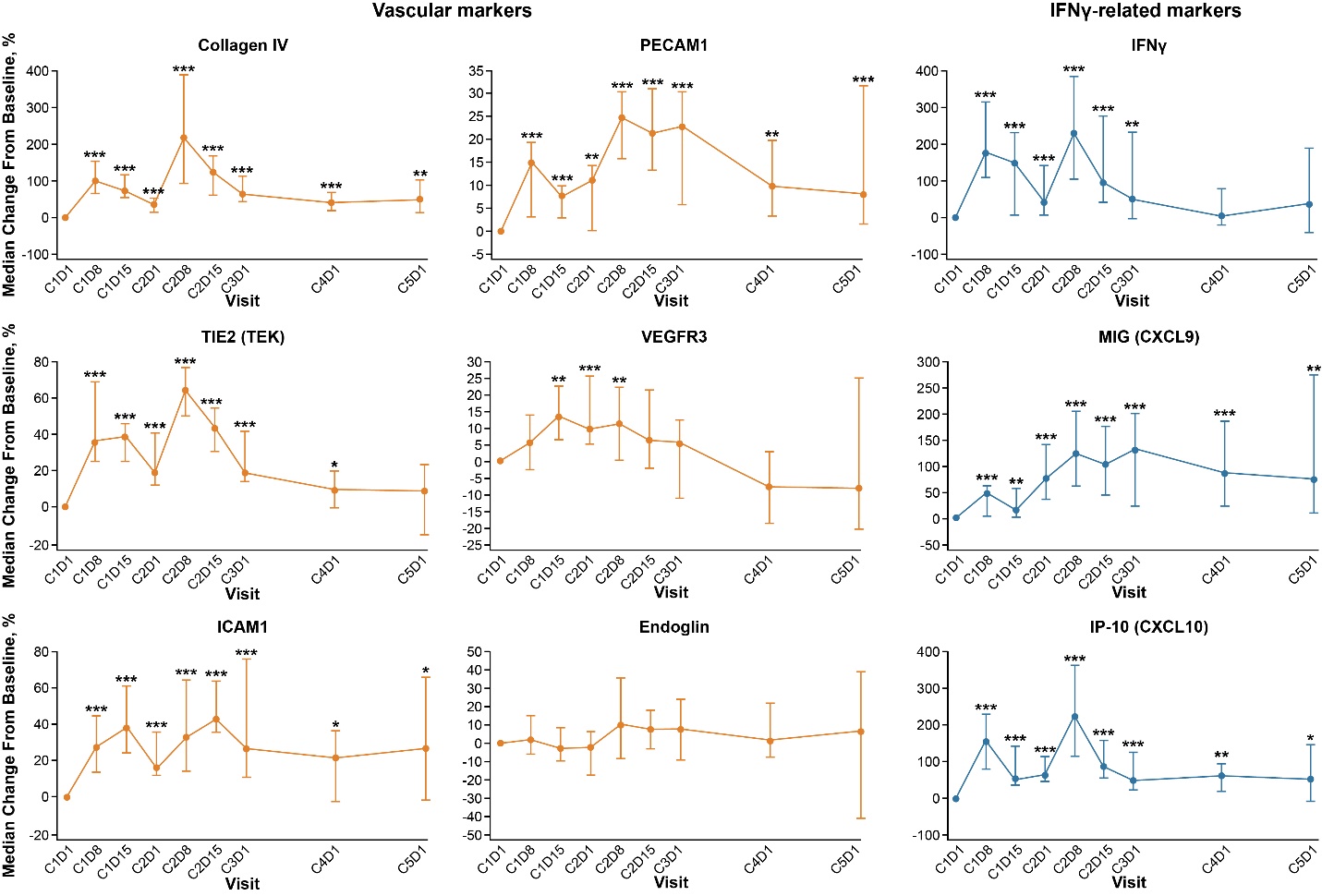


N = 35; Wilcoxon *P*-values: **P* < 0.05; ***P* < 0.01; ****P* < 0.001.

C#D#, cycle #, day #; CXCL, C-X-C motif chemokine ligand ICAM1, intercellular adhesion molecule 1; IFN, interferon; IP10, interferon gamma-induced protein 10; MIG, monokine induced by gamma interferon; PECAM1, platelet endothelial cell adhesion molecule 1; TEK, TEK receptor tyrosine kinase; TIE2, tyrosine kinase immunoglobulin and epidermal growth factor homology domains 2; VEGFR3, vascular endothelial growth factor receptor 3.
